# Occupational scenarios and exposure assessment to formaldehyde: A systematic review

**DOI:** 10.1111/ina.12949

**Published:** 2021-10-27

**Authors:** Vittoria Cammalleri, Roberta Noemi Pocino, Daniela Marotta, Carmela Protano, Federica Sinibaldi, Stefano Simonazzi, Marta Petyx, Sergio Iavicoli, Matteo Vitali

**Affiliations:** ^1^ Department of Public Health and Infectious Diseases University of Rome “La Sapienza” Rome Italy; ^2^ Department of Anatomical Histological Medical Legal Sciences and Locomotor Apparatus University of Rome “La Sapienza” Rome Italy; ^3^ Department of Occupational and Environmental Medicine, Epidemiology and Hygiene INAIL Research Rome Italy

**Keywords:** analytical approach, exposure assessment, exposure levels, formaldehyde, occupational settings

## Abstract

The objectives of the systematic review were to: identify the work sectors at risk for exposure to formaldehyde; investigate the procedures applied to assess occupational exposure; evaluate the reported exposure levels among the different settings. An electronic search of Pubmed, Scopus, Web of Science and ToxNet was carried out for collecting all the articles on the investigated issue published from January 1, 2004 to September 30, 2019. Forty‐three papers were included in the review, and evidenced a great number of occupational scenarios at risk for formaldehyde exposure. All the included studies collected data on formaldehyde exposure levels by a similar approach: environmental and personal sampling followed by chromatographic analyses. Results ranged from not detectable values until to some mg m^−3^ of airborne formaldehyde. The riskiest occupational settings for formaldehyde exposure were the gross anatomy and pathology laboratories, the hairdressing salons and some specific productive settings, such as wooden furniture factories, dairy facilities and fish hatcheries. Notice that formaldehyde, a well‐known carcinogen, was recovered in air at levels higher than outdoor in almost all the studied scenarios/activities; thus, when formaldehyde cannot be removed or substituted, targeted strategies for exposure elimination or mitigation must be adopted.

**Practical Implications**

- The findings of this systematic review provide an overall picture of the worldwide occupational scenarios at potential exposure to formaldehyde and trace evidences for targeted prevention and mitigation actions.
- Personal samplings show higher levels of airborne formaldehyde than environmental ones. Thus, both monitoring modalities should be performed for an accurate risk assessment.

## 1 INTRODUCTION

Formaldehyde is an organic compound that, at room temperature and standard atmospheric pressure, occurs in the form of a colorless, pungent and irritating gas, extremely volatile and highly soluble in water.^1^ It is present as a natural product in many living systems, in the environment, in some foods and in the organism of mammals, including humans, as a product of oxidative metabolism.^2^

Although formaldehyde is naturally present in the troposphere, due to its formation during the oxidation of hydrocarbons,^3^ the main sources determining human exposure are anthropogenic. Among these, some are present in indoor environments such as products containing and releasing formaldehyde (insulating materials, resins, glues, chipboard, plywood, fabrics, etc),^4^ while others are related to activities involving combustion processes, tobacco and e‐cigarettes active and passive smoking and cooking (especially frying).^3^, ^5^ Formaldehyde is a well‐known occupational carcinogen and a recognized sensory irritant compound, especially for sensitive individuals,^6^, ^7^ present in many different working scenarios.^8^ Indeed, formaldehyde is widely used in numerous production processes and sanitary applications due to its chemical‐physical characteristics and broad spectrum microbicide activity.^9^, ^10^ The International Agency for the Research on Cancer (IARC) has identified three main occupational scenarios where workers may be exposed to formaldehyde at air concentrations significantly higher than the indoor and outdoor background levels: (i) the production of formaldehyde and/or its solutions; (ii) the production of products containing formaldehyde or during their use and (iii) the combustion of products generating formaldehyde.^8^ Thus, workers in industrial production processes (resins, plastics, semi‐finished wood products, furnishing accessories and textiles),^11^, ^12^ professionals of gross anatomy and pathology laboratories, veterinarians, embalmers,^9^, ^10^, ^13^, ^14^, ^15^ breeders,^16^ carpenters, industrial launderers,^17^ firefighters, beauticians and printing‐rooms workers^18^, ^19^, ^20^ are the categories at higher risk of exposure to formaldehyde.

In this regard, a robust scientific evidence has highlighted over the years several acute and chronic adverse health effects deriving from such exposure.^21^, ^22^, ^23^, ^24^, ^25^ Moreover, after a revision of the scientific literature, IARC in 2004 has classified formaldehyde as group I carcinogen with sufficient evidence for nasopharyngeal carcinoma^26^ and, afterward, also for leukaemia.^8^ Then, given the evidences, in 2011 the listing status of formaldehyde was changed also from “reasonably anticipated to be a human carcinogen” to “known to be a human carcinogen based on sufficient evidence of carcinogenicity” in the Twelfth Annual Report on Carcinogens of the National Toxicology Program (NTP).^27^ More recently, the European Commission (EC) has reclassified formaldehyde to carcinogenic category 1B (may cause cancer by inhalation) and mutagen category 2 (suspected of causing genetic defects).^28^ Following this reclassification, the EC Regulation No. 1272/2008^29^ on classification, labelling and packaging of substances was amended, and the hazard classification of formaldehyde labelling was modified, as shown in Table 1.

**TABLE 1:** Classification of formaldehyde hazard statements

Hazard categories	Hazard statements
Carc. 1B	H350: may cause cancer by inhalation
Muta. 2	H341: suspected of causing genetic defects
Acute Tox. 3	H301: toxic if swallowed
Acute Tox. 3	H311: toxic in contact with skin
Acute Tox. 3	H331: toxic if inhaled
Skin Corr. 1B	H314: causes severe skin burn and eye damage
Skin Sens. 1	H317: may cause an allergic skin reaction

The classification of formaldehyde as a carcinogen has led to the need to re‐evaluate the risk management systems for potentially exposed workers, as implemented in the various occupational settings. In particular, the typical chemical risk assessment had to move toward a carcinogenic one. This implied the obligation to first evaluate the replacement of formaldehyde with other non‐carcinogenic substances or, if not possible due to technical reasons (often due to cost‐benefit constraints), to mitigate any exposure.

The need to carry out accurate occupational risk assessments for formaldehyde has therefore led to the improvement of sampling and analysis methods. In particular, occupational exposure is usually evaluated by active or passive sampling carried out in fixed positions (environmental sampling) and/or through personal samplers. As regards analysis methods, the airborne formaldehyde can currently be measured at ng m^3^ levels by sampling air with specific sorbent tubes containing 2,4‐dinitrophenyhydrazine or 2‐(hydroxymethyl)piperidine as derivatizer with a built‐in ozone scrubber, and quantifying it by High Performance Liquid Chromatography Mass Spectrometry (HPLC‐MS/MS) or Gas Chromatography Mass Spectrometry (GC‐MS); alternatively, there are portable samplers/analyzers, equipped with photoacoustic spectroscopy detectors or electrochemical detectors, which have a sensitivity of the order of few μg m^3^.

The aim of the present systematic review was to examine the scientific literature reporting experimental data on occupational exposure to formaldehyde from 2004 to the time of the review's conduction (until to September 30, 2019); 2004 was chosen as the first year of classification of formaldehyde as a carcinogen^24^ and, consequently, the year from which the analytical methods used must surely be reliable and sensitive. In particular, specific objectives were to (i) identify the work sectors at risk of exposure to formaldehyde; (ii) investigate the managing procedures used to assess occupational exposure; (iii) evaluate the reported exposure levels among the different settings.

## 2 MATERIALS AND METHODS

### 2.1 Search strategy

This systematic review was performed according to the PRISMA statement.^30^

Three investigators (V.C., D.M. and R.N.P.) searched published studies from January 1, 2004 to September 30, 2019 through the electronic databases MEDLINE via PubMed, SCOPUS and TOXNET. The search terms “(occupational OR workplace OR professional) AND exposure AND formaldehyde” were used. The results obtained by the three different researchers were merged by EndNote X9 software and then all duplicates were removed.

### 2.2 Inclusion/Exclusion criteria

We included all the original studies, published in Italian or English in the fixed period, reporting experimental data obtained directly by the authors on occupational exposure to formaldehyde. In vitro and animal studies as well as all kind of reviews, reports, monographs, book chapters and conference acts were excluded. Then, the title and the abstract of the included articles were independently reviewed by three investigators (V.C., D.M. and R.N.P.). Articles which did not fall within the inclusion criteria were excluded during this phase.

In the following phase, the full text of the remaining potentially eligible papers was independently examined by the same three investigators for final decision on their inclusion or not in the review.

During this multi‐step exclusion process, any disagreement in the decision on the examined studies was discussed until consensus was reached among the three investigators. All the process was supervised by other two different investigators (C.P. and M.V.).

### 2.3 Study quality and evaluation

Study quality was independently assessed by three investigators (V.C., D.M. and R.N.P.) using the Joanna Briggs Institute (JBI) Critical Appraisal Checklist for Analytical Cross‐Sectional Studies.^31^ This tool was used to evaluate the risk of bias through a checklist of eight questions, which include sample selection (two questions), exposure evaluation (two questions), confounding factors (two questions), outcomes and appropriate statistical analysis (two questions). The possible answers for each question were “yes,” “no,” “unclear” and “not applicable.” According to a previously bias assessment that has been already described,^32^ if the answer “yes” was ≥50% of all questions, the evaluated paper was considered with low risk of bias; on the contrary, if the answer “no” was ≥50%, the risk of bias was high. Finally, if the answer “unclear” was ≥50%, the risk of bias was considered uncertain. Studies that presented high or uncertain risk of bias were excluded from the qualitative synthesis of the present review.

All the studies included in the review were synthetized according to year of publication, country, occupational setting, exposure assessment methodology, and main results.

## 3 RESULTS

### 3.1 Study selection process

**FIGURE 1:**
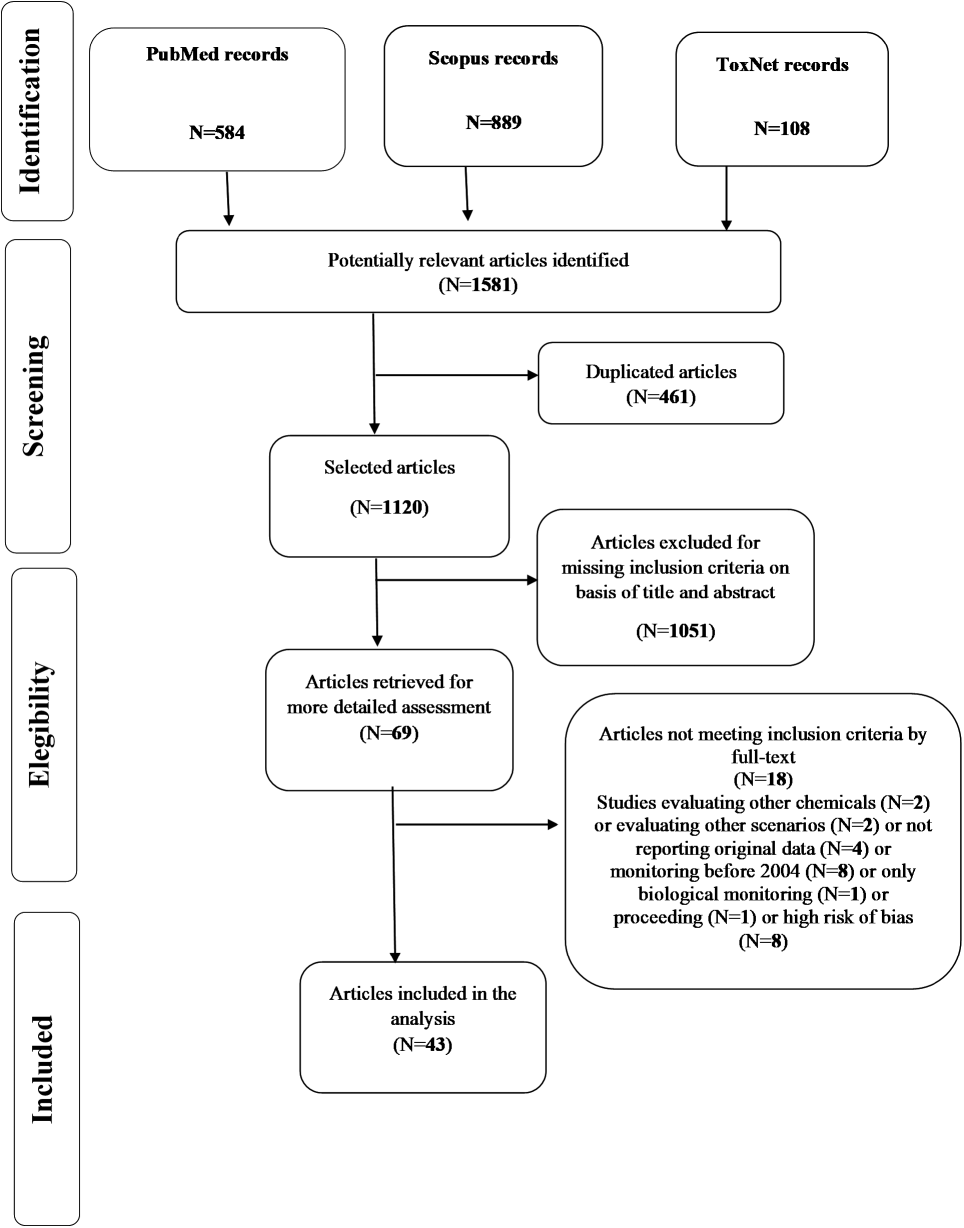
PRISMA flow diagram of the literature search

Figure 1 shows the flow chart summarizing the selection steps for the systematic review.

**FIGURE 2:**
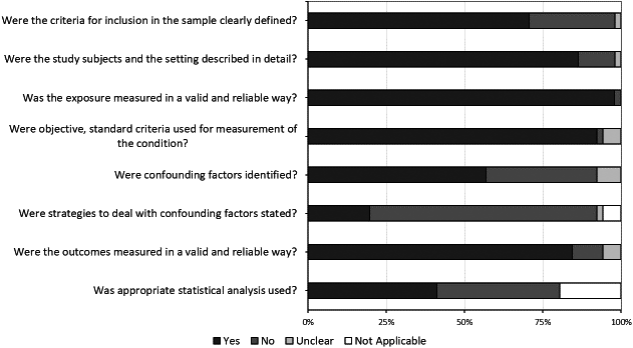
Results of the risk of bias assessment for each question of the checklist

In total, we recovered 1581 studies from all searched databases (584 from PubMed, 889 from Scopus, 108 from ToxNet) and, after removing the 461 duplicates, 1120 articles remained. Out of the remaining 1120 papers, 1051 were excluded after review of their titles and abstracts. Thus, the full‐text of 69 papers were searched and evaluated considering the inclusion/exclusion criteria and the quality assessment. After the evaluation of the full‐text, 18 articles were excluded for the following reasons: 2 articles evaluated the occupational exposure to other chemicals, 2 did not assess occupational exposure, estimated the exposure to formaldehyde based on data reported in national/institutional databases, reported measured performed before the year 2004, 1 just reported data on biological monitoring, 1 was a proceeding.^6^, ^10^, ^11^, ^12^, ^18^, ^33^, ^34^, ^35^, ^36^, ^37^, ^38^, ^39^, ^40^, ^41^, ^42^, ^43^, ^44^, ^45^ In Figure 2 are reported the results of risk of bias assessment for all the 51 articles, considering the percentage of the responses to each question of the checklist.

Finally, 8 articles were excluded because at high risk of bias.^46^, ^47^, ^48^, ^49^, ^50^, ^51^, ^52^, ^53^

At the end of these steps, 43 articles, all with a low risk of bias, were included in the systematic review. In particular, all the papers described the results of cross‐sectional studies performed in several countries worldwide: 12 in USA, 4 in Brazil, 3 in Australia, 3 in Japan, 3 in Taiwan, 3 in Thailand (1 conducted at the same time in Malaysia), 2 in Malaysia (1 conducted at the same time in Thailand), 2 in Portugal, 2 in Italy, 2 in China, 1 in Saudi Arabia, 1 in Iran, 1 in Greece, 1 in Spain, 1 in Egypt, 1 in Sweden and 1 in Korea, all summarized in Tables 2, 3, 4, 5.

**TABLE 2:** Selected characteristics of studies (*n* = 12) involving healthcare and research settings included in the systematic review

First author (Year) Country	Sampling setting and sites	Exposure assessment		Exposure levels
		Analytical methodology (sampling and analysis)	Sampling period	
Cavalcante, (2005)^54^ Brazil	University research institute (offices, classrooms, laboratories, library, print rooms)	Active environmental sampling with 2,4‐DNPH cartridge; HPLC‐UV/DAD	Typical working day	Environmental (min–max) = 0.023–0.162 mg·m^−3^ Personal = ND
Ohmichi (2006)^13^ Japan	Gross anatomy laboratory of a medical school (dissection room)	Passive environmental and personal sampling with 2,4‐DNPH cartridge; HPLC‐UV/VIS	Typical working day in the dissection room (from 1.1 to 6 h)	Environmental (min–max) = 0.27–1.36 mg·m^−3^ Personal (min–max) = 0.40–1.84 mg·m^−3^
Perdelli (2006)^55^ Italy	Pathology departments (offices, laboratories, processing rooms, reduction rooms, storage rooms)	Passive environmental sampling with 2,4‐DNPH cartridge; HPLC‐UV/DAD	Typical working day	Environmental (min–max) = 0.017–2.048 mg·m^−3^ Personal = ND
Lakchayapakor (2010)^56^ Thailand	University gross anatomy laboratory (dissection room)	Active environmental and personal sampling with 2‐hydroxymethyl piperidine cartridge; GC‐FID	Typical working day	Environmental (min–max) = 0.501–0.726 mg·m^−3^ Personal (min–max) = 0.590–1.059 mg·m^−3^
Vohra (2011)^57^ Saudi Arabia	University gross anatomy laboratory (dissection room)	Passive environmental and personal sampling with 2,4‐DNPH cartridge; HPLC‐UV/VIS	Typical working day in the dissection room (from 2 to 3 h)	Environmental (min–max) = 0.66–1.61 mg·m^−3^ Personal (min–max) = 0.77–2.15 mg·m^−3^
Azari (2012)^15^ Iran	University gross anatomy laboratory (indoor laboratory, corridor, moulage, classroom)	Active environmental and personal sampling with 2,4‐DNPH cartridge; HPLC‐UV/VIS	Typical working day in the dissection room (usually 2 h)	Environmental (min–max) = 0.257–0.714 mg·m^−3^ Personal (min–max) = 0.184–1.115 mg·m^−3^
De Ochs (2012) Brazil	University morphology department (corridor, entrance hall, embalming room, anatomy laboratory)	Active environmental and personal sampling with 2,4‐DNPH cartridge; HPLC‐UV/VIS	Typical working day (from 0.5 h in embalming room to 4 h in other sites)	Environmental (min–max) = 0.03–2.52 mg m^−3^ Personal (min–max) = 1.89–4.82 mg m^−3^
Saowakon (2015)^59^ Thailand	University gross anatomy laboratory (dissection room)	Active environmental and personal sampling with 2,4‐DNPH cartridge; HPLC‐UV/VIS	Typical working day in the dissection room (typical dissection sessions)	Environmental (min–max) =0.146–0.518 mg m^−3^ Personal (min–max) =0.157–1.469 mg m^−3^
Bellisario (2016)^60^ Italy	Hospital operating theater	Passive personal sampling with 2,4‐DNPH cartridge; HPLC‐UV/VIS	Typical working day (8 h)	Environmental = ND Personal (mean ± SD) = 0.034 ± 0.008 mg m^−3^
Higashikubo (2017)^61^ Japan	Medical facilities of a prefecture (pathology laboratories, anatomy laboratories, organ preservation facilities, disinfection facilities, dissecting room)	Active environmental sampling with 2,4‐DNPH cartridge; HPLC‐UV/DAD	24 h for pathology laboratory and 10 min for other sites	Environmental (min–max) = <LOD−2.65 mg m^−3^ Personal = ND
Lee (2017)^62^ West Virginia and Texas (USA)	Hospital pathology laboratory and hospital histology laboratory	Active and passive environmental and personal sampling with 2,4‐DNPH cartridge; HPLC‐UV/VIS	Typical working day (117–515 min)	Active environmental (min‐max) = 0.01–0.31 mg m^−3^ Passive environmental (min–max) = 0.02–0.36 mg m^−3^ Active personal (min–max) = 0.01–0.46 mg m^−3^ Passive personal (min–max) = 0.01–2.70 mg m^−3^
Kwong (2018)^63^ Malesia	Learning institute (laboratories, workshops)	Active environmental monitoring with formaldehyde meter monitor (electrochemical sensor)	Typical working day	Environmental (min–max) = 0–1.973 mg m^−3^ Personal = ND

Abbreviations: 2,4‐DNPH, 2,4‐dinitrophenylhydrazine; HPLC, high performance liquid chromatography; GC‐FID, gas chromatography ‐ flame ionization detector; LOD, limit of detection; ND, not determined; UV/DAD, ultraviolet/diode array detector; UV/VIS, ultraviolet/visible detector.

**TABLE 3:** Selected characteristics of studies (*n* = 11) involving esthetic and wellness settings included in the systematic review

First author (Year) Country	Sampling setting and sites	Exposure assessment		Exposure levels
		Analytical methodology	Sampling period	
Tsignonia (2010) Greece	Beauty salons	Active environmental monitoring with chromatometric detector tubes	Typical working day	Environmental = <LOD Personal = ND
Pierce (2011)^66^ Illinois, California (USA)	Hair salon	Active environmental and active and passive personal sampling with 2,4‐DNPH cartridge; HPLC‐UV/VIS Active environmental monitoring with formaldehyde meter monitor (UV detector)	Typical working day	Environmental (min–max) = 0.06–4.48 mg m^−3^ Personal = ND
Huang (2012) Taiwan	Aromatherapy spa	Active environmental sampling with 2,4‐DNPH cartridge; HPLC‐UV/VIS	90–120 min	Environmental (min–max) = 0.026–0.030 mg m^−3^ Personal = ND
Alaves (2013)^70^ Utah (USA)	Nail salons	Active environmental sampling with 2,4‐DNPH cartridge; HPLC‐UV/VIS	Typical working day (8 h)	Environmental (min‐max) = 0.011–0.040 mg m^−3^ Personal = ND
Peteffi (2016)^65^ Brazil	Beauty salons	Passive environmental sampling with 2,4‐DNPH tape; HPLC‐UV/VIS Biological sampling (urine); headspace GC‐FID	Typical working day	Environmental (min–max) = 0.09–0.20 mg m^−3^ Personal = ND Biological (min–max) = 2.72–53.91 mg L^−1^
Aglan (2020) Egypt	Hair salons	Passive environmental sampling with 2,4‐DNPH tape; HPLC‐UV/VIS	15 min	Environmental (mean ± SD) = 2.10 ± 0.34 mg m^−3^ hairstylists involved in hair straightening procedures for <5 years and 2.29 ± 0.20 mg m^−3^ for hairstylists involved in hair straightening procedures for >5 years. Personal = ND
Chang (2018)^69^ Taiwan	Hair salons	Active environmental and personal sampling with 2‐hydroxymethyl piperidine cartridge; GC‐MS	5 h	Environmental (min–max) = 0.012–1.040 mg m^−3^ Personal = 0.295–0.468 mg·m^−3^
Heaton (2019)^71^ Alabama (USA)	Nail salon (experimental chamber)	Active environmental monitoring with formaldehyde meter monitor (electrochemical sensor)	15 min	Environmental (min–max) = 0.15–0.27 mg m^−3^ Personal = ND
Lamplugh (2019)^72^ Colorado (USA)	Nail salons	Active environmental sampling with 2,4‐DNPH cartridge; HPLC‐UV/DAD	8 h	Environmental (min–max) = 0.005–0.021 mg·m^−3^ Personal = ND
Pexe (2019)^66^ Brazil	Beauty and haidresser salons	Active personal sampling with 2,4‐DNPH cartridge; HPLC‐UV/VIS Passive environmental sampling with 2,4‐DNPH tape; HPLC‐UV/VIS	15 min for actives 8 h for passive	Environmental (min–max) (passive) = 0.10–2.40 mg m^−3^ Environmental (min–max) (active) = <LOD−5.15 mg m^−3^ Personal = ND
Zhong (2019)^73^ Michigan (USA)	17 nail salons	Active environmental monitoring with a formaldehyde colorimetric/photoelectric sensor	At least 30 min	Environmental (min–max) = <LOD–0.040 mg·m^−3^ Personal = ND

Abbreviations: 2,4‐DNPH, 2,4‐dinitrophenylhydrazine; HPLC, high performance liquid chromatography; GC‐FID, gas chromatography ‐ flame ionization detector; GC‐MS, gas chromatography ‐ mass spectrometry; LOD, limit of detection; ND, not determined; UV/VIS, ultraviolet/visible detector; UV/DAD, ultraviolet/diode array detector.

**TABLE 4:** Selected characteristics of studies (*n* = 10) involving industrial settings included in the systematic review

First author (Year) Country	Sampling setting and sites	Exposure assessment		Exposure levels
		Analytical methodology	Sampling period	
Lillienberg (2008)^75^ Sweden	Machine shops	Active and passive personal sampling with 2,4‐DNPH cartridge; HPLC‐UV/VIS	6–8 h	Environmental = ND Personal = 0.001–0.154 mg m^−3^
Ratnasingam (2010)^76^ Malaysia, Thailand	Wooden furniture manufacturing factories	Active personal sampling with 2,4‐DNPH cartridge; HPLC (detector not specified)	30 min	Environmental = ND Personal = 2.07–2.72 mg m^−3^
Traviss (2010)^80^ New Hampshire (USA)	Materials recovery facility	Active environmental sampling with 2,4‐DNPH cartridge; HPLC‐UV/VIS	One work shift per fuel type	Environmental (min–max) = about 0.0003‐about 0.0025 mg m^−3^ Personal = ND
Tikuisis (2010)^77^ Canada	Commercial‐scale processing of polyethylene	Active environmental and personal sampling with 2,4‐DNPH cartridge; HPLC‐UV/VIS	Full‐shift workplace	Environmental = <LOD Personal = <LOD
Lee (2012)^78^ Korea	2 tire manufacturing plants	Active environmental sampling with 2,4‐DNPH cartridge; GC‐NPD	Full‐shift workplace	Environmental (min–max) = 0.011–0.036 mg m^−3^ Personal = ND
Teixeira (2013)^79^ Portugal	Wastewater treatment plant	Active environmental monitoring with a formaldehyde colorimetric/photoelectric sensor	30 min	Environmental = always <LOD Personal = ND
Doane (2014)^81^ New York (USA)	Two dairy facilities	Active environmental monitoring with formaldehyde meter monitor (electrochemical sensor)	Three consecutive days	Environmental (min–max) = 0‐about 3.0 mg m^−3^ Personal = ND
Wang (2014)^82^ Taiwan	Decorating workplace during the decorating engineering.	Active environmental monitoring with formaldehyde meter monitor (electrochemical sensor)	5 min	Environmental (min–max) = 0.10 ± 0.03–0.86 ± 0.54 mg m^−3^ Personal = ND
Voorhees (2016)^83^ South Dakota (USA)	Fish hatchery incubation room	Active environmental monitoring with formaldehyde meter monitor (electrochemical sensor)	60 min + additional 30 min if the values were elevated above the basal level	Environmental = <LOD‐about 2.5 mg m^−3^ Personal = ND
Rahman (2017)^84^ New York State (USA)	Manufacturing and storage of wood pellets in a warehouse and enclosed test chambers of wood pellets industry	Active personal sampling with 2‐(hydroxymethyl)piperidine cartridge; GC‐MS	30 min	Environmental = ND Personal (min–max) = 0.16–0.19 mg m^−3^ (drums) and 0.01–0.65 mg m^−3^ (warehouse)

Abbreviations: 2,4‐DNPH, 2,4‐dinitrophenylhydrazine; HPLC, high performance liquid chromatography; GC‐MS, gas chromatography/mass spectrometry; GC‐NPD, gas chromatography/nitrogen phosphorus detectors; LOD, limit of detection; ND, not determined; UV/VIS, ultraviolet/visible detector.

**TABLE 5:** Selected characteristics of studies (*n* = 10) involving fire fighters' and other settings included in the systematic review

First author (Year) Country	Sampling setting and sites	Exposure assessment		Exposure levels
		Analytical methodology	Sampling period	
Baldauf (2006)^88^ North Carolina (USA)	Gardening activities	Active personal sampling with 2,4‐DNPH cartridge; HPLC‐UV/VIS	30 min–2 h	Environmental = ND Personal (min–max) = 0.01‐about 4 mg m^−3^
Pang (2007)^89^ China	29 vehicles including taxi, bus and subway	Passive environmental sampling with 2,4‐DNPH cartridge; HPLC‐UV/VIS	Evening rush hours	Environmental = 0.015–0.094 mg m^−3^ Personal = ND
Reisen (2009)^85^ Australia	Firefighters' exposure to bushfire smoke	Passive personal sampling with 2,4‐DNPH filter paper; HPLC‐UV/VIS Active environmental sampling with 2,4‐DNPH filter cassette; HPLC‐UV/VIS	35–360 min	Environmental (min‐max) = 0.07–0.65 mg m^−3^ Personal (min–max) = 0.07–0.71 mg m^−3^
Reisen (2011)^86^ Australia	Firefighters' exposure to bushfire smoke	Active and passive personal sampling with 2,4‐DNPH filter paper; HPLC‐UV/VIS	‐	Environmental = ND Personal (min–max) = <LOD−0.817 mg m^−3^
Belloc‐Santaliestra (2015)^90^ Spain	Highway tollbooth	Active personal sampling with 2,4‐DNPH filter paper; HPLC‐UV/VIS	7–8 h	Environmental = ND Personal (min–max) = <LOD−0.0162 mg m^−3^
Ceballos (2016)^93^ Ohio (USA)	Four dry cleaning shops	Active environmental and personal sampling with 2‐(hydroxymethyl)piperidine cartridge; GC‐FID	Full‐shift	Environmental (min‐max) = <LOD−0.054 mg m^−3^ Personal (min–max) = <LOD−0.109 mg m^−3^
Ho (2016)^92^ China	University campus (offices, dining room, student dormitory, library, print rooms)	Active environmental sampling with 2,4‐DNPH cartridge; HPLC‐UV/DAD	Typical working day or typical spending time	Environmental (mean ± SD) = 0.009 ± 0.009 mg m^−3^ Personal = ND
Vincente (2016) Portugal	Two copy centers	Active environmental monitoring with formaldehyde meter monitor (electrochemical sensor)	24 h	Environmental (mean) = 0.04 ± 0.01 and 0.03 ± 0.01 mg m^−3^ for copy centers A and B Personal (min–max) = ND
Kirk (2019)^87^ Australia	Compartment fire behavior training	Passive personal sampling with 2,4‐ DNPH tape; HPLC‐UV/VIS Active personal sampling with 2,4‐ DNPH cartridge; HPLC‐UV/VIS	20–35 min for passive personal sampling and 12–18 min for active personal sampling	Environmental (min‐max) = <LOD−0.043 mg m^−3^ Personal (min–max) = <LOD−0.0087 mg m^−3^
Shinohara (2019)^91^ Japan	Gas station	Active environmental and personal sampling with 2,4‐DNPH cartridge; HPLC (detector not specified)	2 h in a spring day and 2 h in a winter day	Environmental (mean) = 0.010 mg m^−3^ (spring) or 0.024 mg m^−3^ (winter) Personal (mean) = 0.005 mg m^−3^ (spring) or 0.012 mg m^−3^ (winter)

Abbreviations: 2,4‐DNPH, 2,4‐dinitrophenylhydrazine; HPLC, high performance liquid chromatography; LOD, limit of detection; ND, not determined; UV/VIS, ultraviolet/visible detector.

All the included articles were grouped according to the studied occupational scenarios as follows: healthcare and research (Table 2), esthetic and wellness (Table 3), industrial (Table 4), fire fighters' and other settings (Table 5).

### 3.2 Main characteristics of the studies involving healthcare and research settings

In Table 2 are reported the main characteristics of the included studies performed in healthcare and research settings.

In total, 12 papers reported the results of studies performed in healthcare and research settings.^13^, ^15^, ^54^, ^55^, ^56^, ^57^, ^58^, ^59^, ^60^, ^61^, ^62^, ^63^ Most of these studies^13^, ^15^, ^55^, ^56^, ^57^, ^58^, ^59^, ^62^ evaluated the occupational exposure to formaldehyde in gross anatomy laboratories (dissection room) and pathology or histology laboratories during a typical working day, with a variable sampling period according to the specific work activities. Other occupational healthcare and research scenarios included different indoor environments of university research institutes^54^ or of learning institute^63^ and hospital operating theaters.^60^ Sampling and analyses were carried out in the greatest part of the studies by the use of active and/or passive environmental sampling and/or active and/or passive personal sampling with 2,4‐DNPH cartridges and HPLC with UV/DAD or UV/VIS. The exceptions to these cases were the studies of Lakchayapakor et al.^56^ and of Kwong et al.^62^ The first study evaluated formaldehyde exposure performing active environmental and personal sampling by the use of 2‐hydroxymethyl piperidine cartridges and GC‐FID technique, while the second performed an active environmental monitoring with a formaldehyde meter monitor.

The results of the included studied recovered exposure values ranging from not detectable levels to concentrations in the order of about 1–3 mg m^−3^, with the highest level recovered in the hospital pathology and histology laboratories (maximum value for passive personal monitoring equal to 2.70 mg m^−3^).^62^ Notice that personal sampling involved higher concentrations compared to workplace sampling in all cases.

### 3.3 Main characteristics of the studies involving esthetic and wellness settings

Table 3 shows the main characteristics of the studies carried out in esthetic and wellness settings.

Eleven papers included in the present review evaluated formaldehyde occupational exposure in esthetic and wellness scenarios, including beauty salons^64^, ^65^, ^66^ hair salons,^67^, ^68^, ^69^ nail salons^70^, ^71^, ^72^, ^73^ and aromatherapy spa.^74^ Similarly, to the monitoring performed in research and healthcare scenarios, even in these settings exposure assessment was performed during a typical working day with variable sampling periods. The main procedures used for assessing formaldehyde occupational exposure were active and/or passive environmental and active and/or passive personal sampling with 2,4‐DNPH cartridges or tapes and HPLC with UV/VIS or UV/DAD detector. In the other cases, the exposure was evaluated by the use of active environmental monitoring with chromatometric detector tubes,^64^ active environmental monitoring with formaldehyde meter monitor,^67^, ^71^ active environmental and personal sampling with 2‐hydroxymethyl piperidine cartridge and GC‐MS,^69^ active environmental monitoring with a formaldehyde colorimetric/photoelectric sensor.^73^ In one study the exposure was also evaluated by the use of biological monitoring.^65^ The levels of environmental formaldehydes ranged from not detectable to more than 4 mg m^−3^, with the highest levels recovered in hair saloons.^66^, ^67^

### 3.4 Main characteristics of the studies involving industrial settings

In Table 4 are reported the studies performed in industrial settings.

Ten studies included in the systematic review were performed in industrial settings, involving different sectors: machine shops,^75^ wooden furniture manufacturing factories,^76^ commercial‐scale processing of polyethylene,^77^ tire manufacturing plants,^78^ a wastewater treatment plant,^79^ a materials recovery facility,^80^ dairy facilities,^81^ decorating workplace during the decorating engineering,^82^ fish hatchery incubation room,^83^ wood pellets industry.^84^ Exposure assessment to formaldehyde was performed with active environmental and active and passive personal sampling with 2,4‐DNPH cartridge and HPLC‐UV/VIS or GC‐MS with NPD, or with active environmental monitoring with formaldehyde meter monitor^79^, ^81^, ^82^, ^83^ or, in one case, by the use of active personal sampling with 2‐(hydroxymethyl)piperidine cartridge and GC‐MS technique.^84^ Exposure levels were in the order of µg m^−3^, but in the cases of wooden furniture manufacturing factories^76^ and fish hatchery incubation room,^83^ exposure concentrations achieves until to 2–3 mg m^−3^.

### 3.5 Main characteristics of the studies involving fire fighters' and other settings

In Table 5 are reported the studies carried out in fire fighters' and other settings and a miscellanea of other occupational scenarios.

As shown in Table 5, three studies^85^, ^86^, ^87^ assessed firefighters' exposure to formaldehyde during their occupational activities. All the studies were performed by the same procedures: active environmental and passive personal sampling with 2,4‐DNPH filters and HPLC with UV/VIS detector. Exposure levels ranged from <LOD to 5 mg m^−3^.

Other monitored occupational activities and/or scenarios included gardening activities,^88^ vehicles such as taxi, bus and subway,^89^ highway tollbooth,^90^ gas station,^91^ school campus as a micro‐scale society,^92^ dry cleaning shops,^93^ copy centers.^94^ In most cases, exposure to formaldehyde was evaluated with active and/or passive environmental and active and/or passive personal sampling with 2,4‐DNPH cartridges and HPLC with UV/VIS or UV/DAD detector. In one study the active environmental monitoring was performed with a formaldehyde meter monitor^94^ while in another study the evaluation was performed by the use of active environmental and personal sampling with 2‐(hydroxymethyl)piperidine cartridges and GC‐FID technique.^93^ The levels of exposure resulted in the order of µg m^−3^, with the exception of the concentrations recovered during garden activities, until to about 4 mg m^−3^.^88^

## 4 DISCUSSION

The present systematic review was conducted to recover scientific evidences on occupational exposure to formaldehyde, in order to define the occupational settings at risk of exposure and the procedures applied to assess exposure levels. These two aims are even more relevant since formaldehyde was recognized as a carcinogen, making mandatory to carry out workers' health surveillance profiled on exposure data.

The first relevant result is related to the great number of occupational scenarios linked to the potential exposure to formaldehyde; indeed, although formaldehyde is a known carcinogen, we found several workplaces and work activities involving a potential exposure to formaldehyde both for its use or its emission during different thermal processes. In total, we grouped four main scenarios: healthcare and research, esthetic and wellness, industrial, firefighters' and a miscellanea of other occupational places/activities.

Healthcare and research settings included gross anatomy, pathology or histology laboratories^13^, ^15^, ^55^, ^56^, ^57^, ^58^, ^59^, ^61^, ^62^ or also operating theaters^60^ and other indoor environments of universities and research or training institutes.^54^, ^63^ In particular, some work activities performed in gross anatomy, pathology or histology laboratories and in operating rooms involve the use of solutions containing formaldehyde for fixing and preserving biological tissues and for preparing cadavers. Thus, formaldehyde vapors can pollute the indoor air of these environments, resulting in a risk of occupational exposure in hospital settings, research laboratories and medical schools. Besides, it is also demonstrated that formaldehyde exposure can occur not only during the handling of formaldehyde and formaldehyde‐ treated materials, but also through inappropriate storage of this substance or treated materials and through an ineffective local exhaust ventilation system.^61^

The second main recognized sector include esthetic and wellness settings, such as beauty, hair or nail salons and spa.^64^, ^65^, ^66^, ^67^, ^68^, ^69^, ^70^, ^71^, ^72^, ^73^, ^74^ In particular, hair dressing activities exposed to higher levels of formaldehyde respect to nail and beauty salons and spas. Probably hair dressers use routinely specific products containing formaldehyde, consciously or because not clearly reported in the label.^68^, ^95^ Likewise, detectable levels of formaldehyde were found also in nail and beauty salons due to its presence in care products used in these settings.

As regard to the industrial scenarios,^75^, ^76^, ^77^, ^78^, ^79^, ^80^, ^81^, ^82^, ^83^, ^84^ airborne formaldehyde is generally present at low levels (micrograms), except where it is directly released from resins^76^ or used for its biocidal properties in dairy facilities^76^, ^81^ and fish hatcheries.^83^

In addition to the well‐known exposure profile of firefighters,^85^, ^86^, ^87^ other investigated scenarios are very diversified, both in terms of settings (school campuses, laundries, copy centers etc)^88^, ^89^, ^90^, ^91^, ^92^, ^93^, ^94^ and in terms of formaldehyde levels (from not detectable to some mg m^−3^).

The methodological approach and the analytical methods are similar in the greatest part of the studies and, thus, their results are comparable. Notice that, in general, the results of personal monitoring are higher than environmental ones. This finding should be taken into account when a survey strategy is planned: both modalities should be performed to define risk levels and elaborate a risk assessment document. For this purpose, a comparison of data obtained with regulatory limits must be carried out. At today, however, United States, EU and others countries have adopted different approaches for the toxicological evaluation of experimental and epidemiological data on formaldehyde exposure and effects resulting in different limit values both for long and short term exposure.

This review presents some limitations. Firstly, several studies only report average or min‐max air levels; secondly, only few studies measured short time or peak air concentrations; besides, the time intervals of the monitoring and the number of samples were different from a study to another. Thus, we could not compare exposure data reported by included articles with actual regulatory limits.

Finally, given the differences of the studies included in this review in terms of exposed populations and procedures used for assessing airborne formaldehyde levels, we decided to review and summarize the results of the selected studies rather than to carry out a formal meta‐analysis. Thus, statistical heterogeneity and publication bias were not assessed.

## 5 CONCLUSIONS

The results of the present review demonstrate that there is a great number of diversified occupational scenarios at risk for formaldehyde exposure. Nevertheless, the monitoring approaches are very similar each other and based on environmental and personal sampling followed by chromatographic analyses, thus allowing data comparison. The settings at higher levels of airborne formaldehyde resulted the gross anatomy and pathology laboratories, the hairdressing salons and some specific productive settings such as wooden furniture factories, dairy facilities and fish hatcheries. However, it is important to highlight that in almost all the studied scenarios/activities, formaldehyde was recovered in air at levels higher than outdoor. Considering that formaldehyde is a well‐known carcinogen, targeted strategies for exposure elimination or mitigation (when formaldehyde cannot be removed or substituted) must be adopted.

## CONFLICT OF INTERESTS

None of the authors declares any conflict of interest.

## AUTHOR CONTRIBUTIONS

Vittoria Cammalleri was involved in data curation, formal analysis, methodology, investigation, validation, and writing the original draft. Roberta Noemi Pocino was involved in data curation, formal analysis, methodology, investigation, and writing the original draft. Daniela Marotta was involved in data curation, formal analysis, investigation, and writing the original draft. Carmela Protano was involved in conceptualization, project administration, validation, writing the original draft, and writing, review and editing. Federica Sinibaldi was involved in data curation, investigation, and writing the original draft. Stefano Simonazzi was involved in formal analysis, and writing the original draft. Marta Petyx was involved in validation, and writing, review and editing. Sergio Iavicoli was involved in validation, and writing, review and editing. Matteo Vitali was involved in conceptualization, data curation, formal analysis, funding acquisition, methodology, project administration, resources, supervision, visualization, writing the original draft, and writing, review and editing.

### PEER REVIEW

The peer review history for this article is available at https://publons.com/publon/10.1111/ina.12949.
